# Sterile cerebrospinal fluid ascites presenting as high SAAG ascites: a case report

**DOI:** 10.1186/s12876-019-1116-8

**Published:** 2019-11-27

**Authors:** Darrick K. Li, Jesse M. Platt, Jessica E. S. Shay, Joseph C. Yarze

**Affiliations:** Division of Gastroenterology, Department of Medicine, Massachusetts General Hospital, 55 Fruit Street, Blake Building, 4th Floor, GI Unit, Boston, MA 02114 USA

**Keywords:** Ascites, Cerebrospinal fluid, Serum ascites albumin gradient, Peritoneovenous shunt, Case report

## Abstract

**Background:**

Cerebrospinal fluid ascites is a rare complication of ventriculoperitoneal shunting and is the result of infection and subsequent peritonitis in the majority of cases. Sterile cerebrospinal fluid ascites in which no known infectious etiology is identified, is even more unusual.

**Case Presentation:**

A 26-year-old female with Loeys-Dietz syndrome and congenital hydrocephalus treated with a ventriculoperitoneal shunt, was evaluated after developing new-onset ascites of unclear etiology after abdominal surgery for repair of an aortic aneurysm requiring multiple therapeutic paracenteses. Her serum ascites albumin gradient (SAAG) was greater than 1.1, suggestive of a portal hypertensive etiology. Gram stain as well as multiple cultures of her ascites fluid were both negative. Extensive investigation including hepatic venous portal gradient measurement and liver biopsy revealed no evidence of hepatic disease or portal hypertension. She was ultimately found to have sterile cerebrospinal fluid ascites which was treated successfully with a peritoneovenous shunt.

**Conclusion:**

Sterile cerebrospinal fluid ascites is a rare clinical entity that has only been reported approximately 50 times in the medical literature. In this report, we also highlight it as a rare cause of high SAAG ascites. Moreover, we describe the use of a peritoneovenous shunt as a novel therapeutic option in the management of this condition.

## Background

Ascites is a common clinical problem for which hepatic cirrhosis represents ~ 80% of all cases, while non-hepatic causes including peritoneal malignancy (12%), cardiac failure (5%), and peritoneal tuberculosis (2%) constitute most of the remaining etiologies [1]. A thorough evaluation, including assessment of the serum ascites albumin gradient (SAAG), is crucial to identifying the cause of the ascites and implementing an appropriate course of treatment. Low SAAG (< 1.1 g/dL) ascites is most frequently caused by peritoneal malignancy or tuberculosis and can also be caused by nephrotic syndrome, pancreatic ascites, or protein-losing enteropathy. A SAAG of ≥1.1 g/dL has been shown to be effective at identifying patients with ascites secondary to sinusoidal portal hypertension. Interestingly, the SAAG has been shown to correlate with hepatic sinusoidal pressure and the threshold of 1.1 g/dL roughly corresponds to a portal pressure gradient of 12 mmHg [2], the pressure that is thought to be necessary for the development of ascites in patients with cirrhosis. High SAAG ascites can also be the result of other disease processes including cardiac failure, alcoholic hepatitis, and Budd-Chiari syndrome. In this report, we describe a case of high SAAG ascites secondary to sterile cerebrospinal fluid accumulation.

## Case presentation

**Fig. 1:**
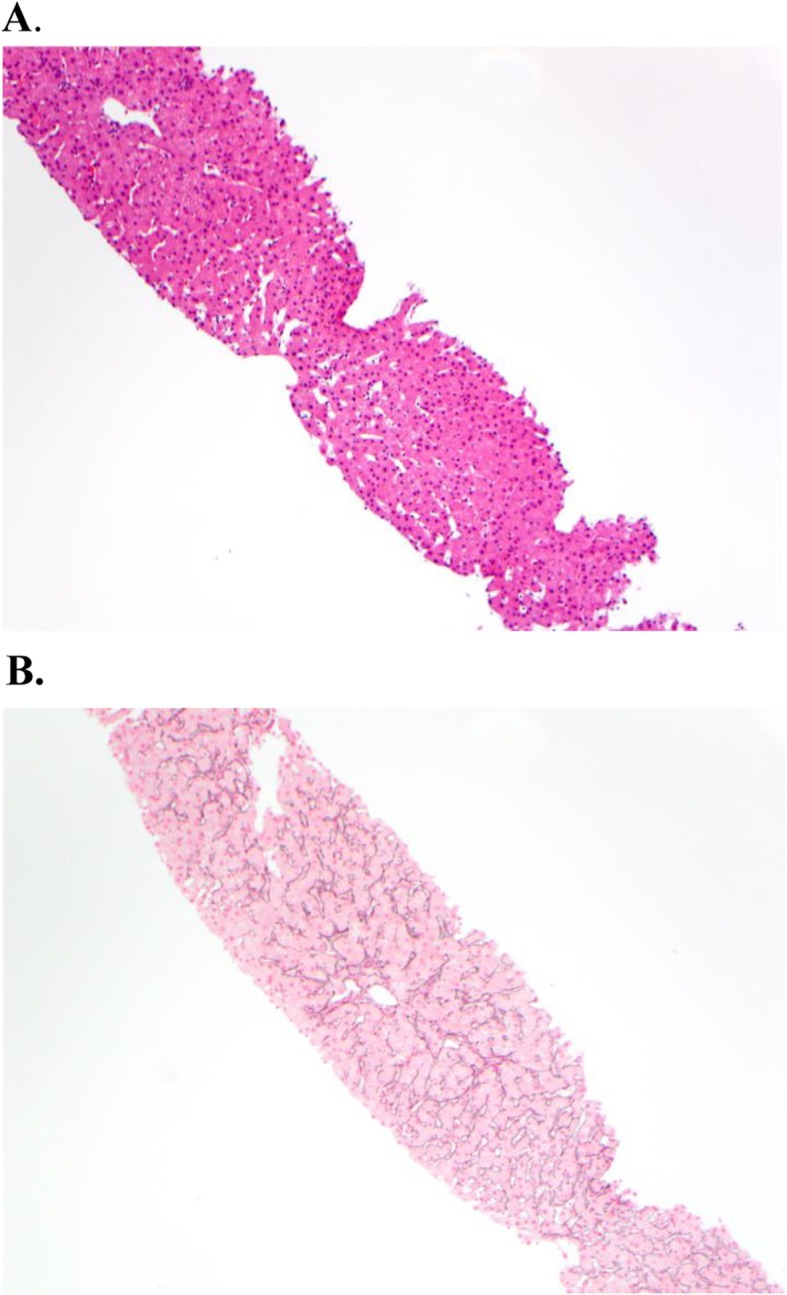
Liver biopsy specimen showing largely normal hepatic parenchyma with mild sinusoidal dilatation with (**a**) hemotoxylin and eosin staining (10x magnification) or (**b**) reticulin staining (10x magnification)

A 26-year-old female with Loeys-Dietz syndrome complicated by multiple aortic aneurysms and aortic dissection, congenital hydrocephalus treated with a ventriculoperitoneal (VP) shunt, was evaluated after developing new-onset ascites of unclear etiology. She had recently undergone resection and grafting of a thoracoabdominal aortic aneurysm with multiple vascular bypasses complicated by bowel wall edema and injury to her inferior vena cava necessitating chest and abdominal washouts. On post-operative day ten, she developed abdominal distension. Physical examination revealed a thin, young woman with multiple abdominal incisions that were stapled, clean, dry and intact without evidence of erythema, purulent drainage or infection. Her abdomen was distended, with bulging flanks and evidence of shifting dullness. CT of her abdomen and pelvis revealed massive ascites and diffuse anasarca. Paracentesis was performed and three liters of straw-colored fluid was removed. Analysis of ascitic fluid revealed 225 WBCs/mm^3^ (60% neutrophils) with total protein 2.0 g/dL, albumin 1.1 g/dL, and SAAG of 1.5. Bacterial, viral, fungal, and mycobacterial cultures of the ascites were negative. Given the elevated SAAG, there was initial concern for underlying hepatic disease and portal hypertension. Liver biochemical tests, complete blood count, and urinalysis were normal. Doppler interrogation of the hepatic vasculature and echocardiography were normal. A shunt study of the ventriculoperitoneal shunt revealed a normally functioning shunt. Invasive hepatic venous pressure measurements were performed. This revealed a right atrial pressure of 6 mmHg, free hepatic vein pressure of 8 mmHg and a wedged hepatic vein pressure of 10 mmHg for a hepatic venous portal gradient of 2 mmHg (normal < 5 mmHg), demonstrating an absence of portal hypertension. Liver biopsy revealed hepatic parenchyma with mild sinusoidal dilation but without inflammation, steatosis, or fibrosis. A reticulin stain revealed mild irregularity of hepatic cords and focal nodularity but this was not felt to be diagnostic of nodular regenerative hyperplasia (Fig. 1). Given the results of the evaluation, a diagnosis of cerebrospinal fluid (CSF) ascites was made, possibly related to decreased peritoneal absorption in the setting of post-operative peritoneal inflammation. A peritoneovenous (Denver) shunt was successfully placed into the right internal jugular vein. Initially, her ascites remained persistent and there was concern regarding limited drainage through the Denver shunt. Conversion of her existing VP shunt to a ventriculoatrial (VA) shunt was considered but given her underlying genetic vasculopathy and potential cardiac risks of another procedure, this was deferred. The Denver shunt was evaluated and was deemed to be functioning properly. Over the next several weeks, her ascites gradually resolved and she required no further therapeutic paracenteses.

## Discussion and conclusions

The SAAG is widely used to help elucidate the etiology of ascites. Ascites due to portal hypertension is characterized by a SAAG of ≥1.1 g/dL, and the ascitic fluid total protein is subsequently used to further differentiate the various causes of ascites in patients with a high SAAG [3]. Cirrhosis represents the most common cause of high SAAG ascites, but constrictive pericarditis, congestive heart failure, Budd-Chiari syndrome, and alcoholic hepatitis are also diagnostic considerations. Sterile CSF ascites is a rare complication of ventriculoperitoneal shunting and to date, there have been approximately 50 cases reported in the English literature. Of these, only four, including our own, report either a paired serum albumin and ascites albumin or a SAAG [4–6] (Table 1). In each of these cases, the SAAG is also reported to be ≥1.1 g/dL. Our case corroborates that CSF ascites is within the differential diagnosis of high SAAG ascites. The pathophysiology of the high SAAG in patients with sterile CSF ascites remains speculative but likely reflects the inherently low albumin content in sterile CSF fluid as a result of the blood-CSF barrier and the absence of de novo albumin synthesis in the central nervous system [7].

**Table 1:** Published cases of CSF ascites with reported SAAG values

Case	Age	Predisposing pathology for CSF ascites formation	SAAG (g/dL)	Treatment
This case	26 years	Abdominal surgery	1.5	Denver shunt, paracenteses
Longstreth and Buckwalter [4]	28 years	Inflammatory reaction to silicone tubing	2.5	Ventriculoatrial shunt
Das et al. [5]	7 years	Chemotherapy	High (not specified)	Ventriculoatrial shunt
Comba et al. [6]	6 years	Unknown	2.75	Ventriculoatrial shunt

Loeys-Dietz syndrome (LDS) is an autosomal dominant connective tissue disorder first described in 2005, characterized by aortic aneurysms, generalized arterial tortuosity, hypertelorism, and cleft palate. LDS has been described due to mutations in the transforming growth factor β I (*TGFBR1*), transforming growth factor β II (*TGFBR2*), transforming growth factor β 2 ligand gene (*TGFB2*), and the decapentaplegic homolog 3 (*SMAD3*) [8]. Clinical complications of LDS are primarily a result of vascular complications including aortic dissection and aneurysmal rupture, which often require aortic surgery as was the case in our patients. Gastrointestinal complications including constipation as well as higher prevalence of eosinophilic gastrointestinal disease have been reported, but hepatic complications specifically related to LDS have not been reported.

Our case is also notable for the successful use of a Denver shunt in the management of CSF ascites, which has not been previously reported. Denver shunts have been successfully used in the management of portal hypertensive and malignant ascites and rely on the principle that ascites flows down a pressure gradient from the peritoneal cavity into the central venous circulation [9, 10]. Backflow of blood is prevented through the use of a one-way valve chamber that lies in the subcutaneous tissue that can be compressed to promote flow and avoid blockage. Complications include infection, blockage, venous thrombosis, and disseminated intravascular coagulation. In the literature, CSF ascites has been treated primarily with conversion of the existing VP shunt to a VA shunt [4]. Our case demonstrates that the use of a Denver shunt may be a safe and viable alternative treatment modality to VA shunting for this condition.

In this report, we characterize sterile cerebrospinal fluid ascites as a rare cause of high SAAG ascites, highlighting the need for clinician awareness for this rare entity in the differential diagnosis of ascites. Moreover, we describe the use of a peritoneovenous shunt for the first time as a novel and viable therapeutic option in the management of this condition.

## Data Availability

Data sharing is not applicable to this article as no datasets were generated or analyzed during the current study.

## Abbreviations

CSF: Cerebrospinal fluid
SAAG: Serum ascites albumin gradient
VA: Ventriculoatrial
VP: Ventriculoperitoneal
